# Food-dependent, exercise-induced gastrointestinal distress

**DOI:** 10.1186/1550-2783-8-12

**Published:** 2011-09-28

**Authors:** Erick Prado de Oliveira, Roberto Carlos Burini

**Affiliations:** 1 Centre for Physical Exercise and Nutrition Metabolism, UNESP School of Medicine, Public Health Department, Botucatu City, São Paulo State, Brazil; 2 Department of Pathology, UNESP School of Medicine, Brazil

**Keywords:** diet, gastrointestinal distress, physical exercise

## Abstract

Among athletes strenuous exercise, dehydration and gastric emptying (GE) delay are the main causes of gastrointestinal (GI) complaints, whereas gut ischemia is the main cause of their nausea, vomiting, abdominal pain and (blood) diarrhea. Additionally any factor that limits sweat evaporation, such as a hot and humid environment and/or body dehydration, has profound effects on muscle glycogen depletion and risk for heat illness. A serious underperfusion of the gut often leads to mucosal damage and enhanced permeability so as to hide blood loss, microbiota invasion (or endotoxemia) and food-born allergen absorption (with anaphylaxis). The goal of exercise rehydration is to intake more fluid orally than what is being lost in sweat. Sports drinks provide the addition of sodium and carbohydrates to assist with intestinal absorption of water and muscle-glycogen replenishment, respectively. However GE is proportionally slowed by carbohydrate-rich (hyperosmolar) solutions. On the other hand, in order to prevent hyponatremia, avoiding overhydration is recommended. Caregiver's responsibility would be to inform athletes about potential dangers of drinking too much water and also advise them to refrain from using hypertonic fluid replacements.

## 1. Introduction

The importance of physical activity to well-being cannot be overstated. The physiological, psychological, and social benefits of regular exercise are plentiful and profound. Examples of such benefits include positive effects on weight, bone strength, metabolic factors (such as glucose and cholesterol), organ function, sleep, mood and self-image. Coupled with the proliferation of team sports and increased choices for individual exercise, the fitness movement has created an increased demand for the care of athletes. Anyone who participates in physical exercise is at risk for injury and illness arising from such activity [1,2].

Strenuous exercise and dehydrated states would be the causes of gastrointestinal symptoms. Gut ischemia would be the main cause of nausea, vomiting, abdominal pain and (bloody) diarrhea [3]. Moreover, anaphylaxis is observed during or soon after exercise when preceded by the intake of a causal food allergen [4,5]. Adequate meal composition and hydration are essential for the prevention of these events.

## 2. Exercise-induced gastrointestinal complaints

There is a very high prevalence of gastrointestinal (GI) complaints during exercise among long-distance runners, triathletes and athletes involved in other types of strenuous long-lasting exercise [6]. These GI complaints occur because of the redistribution of the blood flow, that is shunted from the viscera to skeletal muscle, heart, lung and brain [7].

The symptoms include dizziness, nausea, stomach or intestinal clamps, vomiting and diarrhea. Prevalence of 30-50% has been reported among marathon runners. Severe symptoms include vomiting and diarrhea and occur mainly during running [8]. It has been suggested that these problems occur mainly because of the movements of the gut [9]. However, an association was reported between nutritional practices and GI complaints during a half ironman-distance triathlon with the intake of fiber, fat, protein and concentrated carbohydrate solutions during the triathlon, in particular beverages with very high osmolarity [10].

The symptoms are often mild and may not even affect performance. Some of the symptoms, however, can be life-threatening, such as blood loss in feces in the hours following the running presented by some marathoners and long-distance triathletes [8].

Damage to the gut and impaired gut function is associated with increased of intestinal permeability after a marathon [11]. Moreover, vigorous exercise (jogging, aerobics, dancing, tennis, bicycling, racquetball, swimming, and skiing) [12,13] facilities allergen absorption from the GI tract [14], leading to a food-dependent exercise induces anaphylaxis (FDEIA).

FDEIA is a subtype of anaphylaxis induced by exercise that is related to the intake of specific foods [15]. Allergic symptoms are elicited when triggering factors such as exercise or aspirin intake are added after intake of the causative food [16]. FDEIA is a unique disorder caused by exercise after food ingestion [17].

Ingestion of aspirin combined with exercise increased GI permeability in humans, thus allowing for the detection of food-derived allergens in serum [5]. When food intake and exercise are exposed independently, patients will not experience allergic symptoms [14]. However, the onset of anaphylaxis occurs during or soon after exercise when preceded by the ingestion of a causal food allergen [4,5].

FDEIA is an IgE-mediated hypersensitivity. As in other allergic syndromes, mast cells seem to play a prominent role, and most FDEIA symptoms can be explained based on the release of mast cell mediators, including histamine, leukotrienes (LCT4), and prostaglandins (PGD2) [14,16,18,19]. Increased norepinephrine may be involved in the onset of FDEIA since it may selectively inhibit T-helper (Th) functions while favoring Th-2 responses [20].

Many kinds of food have been identified as causes of FDEIA, but any kind of food appears to be responsible for it. Specific FDEIA has been associated with cereals, seafood, peanut, free nuts, eggs, milk and vegetables [21]. FDEIA only occurs after consumption of a food allergen if this is followed by vigorous physical activity within a few hours of consumption [15]. Elicitation of the allergic symptoms is known to be dependent on the amount of the food intake [16]. FDEIA can be controlled by avoidance of food before exercise [13].

GI problems, hyperthermia and hyponatremia are potentially life-threatening in longer triathlon events. Problems with hyperthermia seem to be related to the intake of highly concentrated carbohydrate solutions, or hyperosmotic drinks, and the intake of fiber, fat and protein [8]. Hyponatremia has occasionally been reported, especially among slow competitors in triathlons, and probably arises from the loss of sodium in sweat in association with very high intake (8-10L) of water or other low-sodium drinks [8].

## 3. Exercise-induced dehydration

During exercise, activity in the sympathoadrenal neuroendocrine system and its plasma hormones increases. Such increase is of major importance for cardiovascular adaptation, thermoregulation and energy-yielding substrate in exercise. Cardiac frequency and contraction force are enhanced; the tone of arterioles in the splanchnic area, kidney and non-contracting muscles and veins is increased, and the spleen is brought to contract. In this way, cardiac output is enhanced, and blood volume and flow are redistributed in favor of the skin and the working muscle [22].

Prolonged exercise at high intensities leads to a quantitative redistribution of blood flow to the exercising muscle (exercise hyperthermia) in proportion to its energy demands of oxygen and substrates. Sympathoadrenal activity, however, reduces water and sodium loss during exercise by decreasing renal blood flow and changing its distribution by direct tubular effects. Moreover, it decreases potassium loss by facilitating its muscular uptake [22].

Blood flow to the skin is increased to facilitate heat dissipation, and sweating implies loss of water and electrolytes from the body. Dehydration of approximately 2-3% of body mass routinely occurs during intermittent high-intensity exercise, especially when the ambient temperature is high. Usually, thirst is triggered when the individual is already 5% dehydrated [23]. The dehydrated state can be worsened by catecholamine-induced thirst suppression [24].

Fluid loss results in decreasing circulatory blood volume, blood pressure, sweat production and stroke volume, as result, vascular resistance increase leading to a skin blood flow decreased, all of which impair heat dissipation. Heart rate rises to some additional 3-5 beats/minute for every 1% body weight loss due to dehydration [25].

Dehydration has a negative effect on endurance performance by increasing muscular glycogen degradation and plasma lactate levels and by causing cardiovascular drift and reduced ability to transport heat to the periphery for dissipation, thus resulting in increased core temperature [26].

### 3.1 Exercise-dependent, dehydration-induced hyperthermia

Heat production during exercise is 15-20 times greater than at rest, and it is sufficient to raise core body temperature by 1°C every 5 minutes if there are no thermoregulatory adjustments [25]. The body's multiple mechanisms for heat dissipation to prevent significant hyperthermia include conduction, convection, evaporation and radiation. As ambient temperature rises above 20°C, the contributions of conduction, convection and particularly radiation, become increasingly insignificant with the bulk of the heat dissipation during exercise resulting from evaporation as sweat. In hot, dry conditions, evaporation may account for as much as 98% of dissipated heat. Sweat evaporation leads to dehydration, which increases body temperature [25].

Any factor that limits evaporation, such as high humidity or dehydration will have profound effects on physiological function, athletic performance, and risk for heat illness [27]. There are five common types of heat illness, the milder forms including heat edema, heat cramps, heat syncope, and heat exhaustion. The most severe form of heat illness is heat stroke [28].

The milder forms of heat illness are widely underreported and underdiagnosed [25]. The symptoms go from none to fatigue, mental confusion, nausea, and vomiting with the signs beginning with peripheral edema developing to muscular spasm, loss of consciousness, hypotension and elevated core temperature up to 40.5°C [25].

Heat stroke is defined as a condition in which body temperature is elevated to a level that causes damage to body tissues, giving rise to a characteristic clinical and pathological syndrome that affects multiple organs [29]. Distinguishing features of heat stroke are marked core body temperature elevations greater than 40.5°C, failing sweating mechanisms, often complete cessation of sweating, and moderate to severe mental status impairment. It is a medical emergency in which total thermoregulatory failure will not reverse without external cooling measures and the mortality rates may exceed 10% [25].

### 3.2 Exercise-dependent dehydration-induced ischemia

Blood flow to central tissues (gut and liver) is reduced during exercise by almost 80%, at 70% of VO_2max _[7]. Such decreased splanchnic blood flow and oxygen supply may induce changes in nutrient absorption, motility and the mucosal integrity of the GI tract, resulting in GI complaints [30]. GI distress has been reported to be common among 30%-50% of endurance athletes, especially during marathons, triathlons and other endurance events. The symptoms seem to occur more often during competition in a warm environment [30] in the presence of systemic dehydration and lower plasma volume [8]. Long-lasting high-dose creatine supplementation (80 g/day during four months) is reported to lead to acute renal failure when associated with exhausting strength exercises and related lower plasma volume [31]. However, few or no adverse effects are observed when taking the recommended dose of creatine (10 g/day) [32,33].

#### 3.2.1 Exercise-induced gastric emptying delay

Gastric emptying (GE) is thought to be negatively affected as exercise intensities reach over 70% VO_2max _[34]. The presence of dehydration in strenuous exercise in cyclists was shown to induce significantly increased nausea, epigastric cramps and delay in gastric emptying. Gastric emptying (GE) was significantly associated with increase in exercise-induced nausea. Exercise by itself led to significant increase in plasma vasopressin and rectal temperature and significant decrease in plasma volume, irrespective of the dehydration state, but vasopressin concentration was significantly higher in dehydrated athletes. By adding dehydration to strenuous cycling, there was a delayed gastric emptying, but no differences in orocecal transit time, intestinal permeability or glucose uptake [30].

In an endurance running experiment, GI complaints were reported only with the dehydration exercise combination without any GI disturbances being reported by athletes in either exercise or dehydration test alone. Dehydration-exercise resulted in slower GE than in other two treatments with the effects of dehydration and exercise being additives in delayed GE. It was concluded that the higher prevalence of GI disturbances may be related to delayed GE caused by exercise-induced dehydration and/or thermal effects [35].

### 3.3 Exercise-dependent ischemia-induced GI distress

Serious gut underperfusion often leads to shock-induced mucosal damage and invasion of gram-negative intestinal bacteria and/or their toxic constituents (endotoxins) into the blood circulation [36]. Elevated plasma endotoxin concentrations were found in 81% of ultramarathoners (90 km), with 2% presenting extremely high values [37].

Reduced GI blood flow induced by strenuous exercise makes the gut mucosa susceptible to ischemic injury, increases mucosa permeability and enhances hidden blood loss, as well as the translocation of protective microbiota and endotoxin generation. It is known that mucosal ischemia depletes cellular ATP leading to cell death and mucosal inflammation [11,38]. Hence, strenuous exercise and dehydration states would be the causes of GI symptoms reported by 70% of athletes, and gut ischemia would be the main cause of nausea, vomiting, abdominal pain and (blood) diarrhea [3].

In an extensive literature review using an evidence-based approach, the risk factors for exercise-induced GI tract symptoms were dehydration (body weight loss > 4% during or after exercise), being a female, younger age, high-intensity exercise, vertical impact sports and medicine use. Poor conditioning, dietary factors and previous abdominal surgery are risk factors with weak evidence that was not well supported [39].

## 4. Exercise-dependent rehydration

Rapid fluid delivery from beverages intake is the goal of oral rehydration solutions and sports drinks [40]. The goal of fluid intake is to consume more fluid orally than it is being lost in sweat. Extracellular fluid rehydration is best achieved with smaller fluid volumes and isotonic sodium solutions. Intracellular rehydration is best achieved with higher volumes and lower sodium (hypotonic) solutions. Hemodynamic responses (the optimization of cardiac output as estimated by heart rate and stroke volume) are similar with 100% or 150% fluid replacement and with hypotonic and isotonic solutions. The addition of sodium and carbohydrates assists with intestinal absorption of water and permits more efficient fluid replacement than water alone [2].

### 4.1 Fluid volume

The maximum rate of intestinal absorption is 0.5 L/hour when cycling at 85% VO_2max _[8]. It was estimated that ~ 0.9L remained in the stomach and intestine at the end of exercise, and subjects complained about abdominal fullness. The intake of large volumes may not be advantageous [8], because no enhance in performance is observed [41,42].

Fluid delivery during exercise represents the integration of GE and intestinal absorption. GE of liquids is regulated by the interaction of gastric volume and feedback inhibition, including nutrient-induced duodenal feedback. This occurs in such a way that the GE rate at any given moment represents the balance between feedback inhibition from the small intestine and the stimulatory effect of gastric volume. It is this balance that is responsible for the inverse relationship between beverage CHO content and GE rate [43].

Fluids empty from the stomach in an exponential manner with an initial rapid emptying phase. In fact, one of the major stimulants of GE is the volume in the stomach with a positive relationship between stomach volume and rate of emptying from the stomach. The absorption of water in the intestine is primarily passive, where water passes across the intestinal membrane due to an osmotic gradient [8].

### 4.2 Fluid composition

In order to determine the effect of osmolality on intestinal (duodenum and/or jejunum) fluid absorption of an orally fluid-replacement beverage intake containing 6% carbohydrate, Gisolfi et al (1998) [44] formulated groups of fluid replacement as hypo, iso or hypertonic with water as placebo. Fluid absorption was given during 85 min of cycling exercise (63.3% VO_2max_) in a mild environment (22°C). There were no differences between groups in GE, total fluid absorption, urine production or plasma volume variations. Water was absorbed faster from the duodenum than the jejunum. It was concluded that osmolality has only a modest effect on gastric emptying and that total fluid absorption of 6% CHO-beverage from the duodenum/jejunum during exercise, within 197-414 osmotic range, is not different from that of water.

The effectiveness of different carbohydrate solutions in restoring fluid balance in situations of voluntary fluid intake was examined in 1.99% body mass dehydrated (intermittent route) subjects [26]. Beginning 30 min after cessation of exercise, the subjects drank *ad libitum *for a period of 120 minutes. Drinks contained 31 mmol/L sodium as NaCl and either 0%, or 2% or 10% glucose, with osmolality of 74,188 and 654 mosm/kg respectively. No differences were observed in total fluid intake, urine output, net fluid balance or in the fraction of the drink intake retained. The authors concluded that in situations of voluntary fluid intake, hypertonic carbohydrate-electrolyte solutions are as effective as hypotonic carbohydrate-electrolyte solutions at restoring whole-body fluid balance [26].

Glucose is actively transported across the intestinal membrane, a process aided by the inclusion of sodium. Water co-transportation during this process is controversial; nevertheless, the addition of sodium and CHO to sports drinks is widely recommended to enhance water absorption [8]. The risks of exercise-induced fluid and electrolyte balance are considerably minimized if oral replacement products are used. If activity is prolonged beyond 60 minutes, then CHO sources and potassium should also be included in the ingested fluid [2].

During competition, optimal CHO concentration seems to be in the range of 5-8%, and athletes should aim to achieve a CHO intake of 60-70 g/hour. Athletes should attempt to limit body mass loss to 1% of body mass. In all cases, a drink should contain sodium (30-50 mmol/L) for optimal absorption and hyponatremia prevention. Post-exercise rehydration is best achieved by consuming beverages that have high sodium content (>60 mmol) in a volume equivalent to 150% of body mass loss [8].

There is convincing evidence that the limitation of 1.0-1.1 g/minute CHO oxidation is not at the muscular level, but most likely located in the intestine or the liver. Intestinal perfusion studies suggest that the capacity to absorb glucose is only slightly in excess of the observed entrance of glucose into the blood, and the absorption rate may thus be a factor that contributes to the limitations. The liver, however, may play an additional important role in that it provides glucose to the blood stream at a rate of only 1.0-1.3 g/min by balancing glucose from the gut and from glycogenolysis/gluconeogenesis. It is possible that when large amounts of glucose are ingested, absorption is a limiting factor, and the liver will retain some glucose and will thus act as a second limiting factor to exogenous CHO oxidation [8].

More recently, advice has been given for athletes engaged in moderate- intensity prolonged exercise to increase CHO intake in the form of multiple transportable carbohydrates (glucose plus fructose) to a rate as high as 90 g/hour (or 1.5 g/min), and this has been shown to increase exogenous CHO oxidation above a single CHO [43]. Furthermore, the intake of a glucose-fructose combined solution increases GE and fluid delivery when compared with a glucose-only solution. Additionally, the combined sugar attenuates heart-rate increase and results in lower rates of perceived exertion and lower loss of body weight than glucose alone or water [43]. Moreover, a solution intake with 1.2 g/min of maltodextrin + 0.6 g/min of fructose show higher carbohydrate oxidation (approximately 1.5 g/min) than 1.8 g/min of maltodextrin (alone) [45].

The effects of increasing carbohydrate (0%, 3%, 6% and 9%) and sodium (0, 20, 40, 60 mmol/L) content upon fluid delivery (using deuterium oxide water) were studied in healthy male seated (twenty-four) subjects. It was concluded that increasing the amount of sodium in a 6% glucose beverage did not lead to increases in fluid delivery and that fluid delivery was compromised when the carbohydrate beverage was increased above 6% [40].

When glucose is used as the CHO source, its concentration is limited to < 2.5% since higher concentrations may delay GE and fluid absorption. In general, the combination of different CHO sources should be > 5% to provide sufficient fuel for the maintenance of muscle performance during activity. However, total CHO concentrations are limited to < 10% since higher CHO content is associated with increased risk for GI distress (abdominal cramps, diarrhea and nausea) owing to the high osmolar load [2]. Hypertonic solutions tend to delay water absorption in the intestine as water instead flows into the intestine to dilute the solution before water is absorbed [8].

Additionally, there is contention as to whether hypertonic solutions reduce the GE rate [46]. However, energy density is considered to be more important in determining GE when solutions with an osmolality close to those normally found in sports drinks are used [8].

The rate of fluid absorptions is closely related to the CHO content of drinks with high CHO concentrations, thus compromising fluid delivery. Hence, a balance must be met between the goal of maintaining hydration status and providing CHO to the working muscle [8]. Slowed gastric emptying associated with high-intensity exercise is further slowed by the consumption of hypertonic carbohydrate beverages, usually given after running [38].

## 5. Exercise-dependent food-induced distress

Gastric emptying is proportionally slowed as the concentration of carbohydrates increases in replacement fluid because of hyperosmolar effects [2].

Current nutritional recommendations to endurance athletes are generally based on advice to: 1) drink during exercise to prevent excessive dehydration and excessive changes in electrolyte balance and; 2) maintain carbohydrate oxidation rates and plasma glucose concentrations. However, these two aims (fluid delivery and carbohydrate delivery) can be difficult to reconcile as increasing the CHO content of a beverage to high levels increases the CHO delivery rate, but decreases fluid delivery. As a compromise between CHO and fluid delivery, it is often recommended that sports drinks have CHO concentrations below 8% [43].

### 5.1 Hyponatremia

Electrolyte imbalance which is commonly referred to as "water intoxication" and results from hyponatremia (low plasma sodium) due to excessive water intake has occasionally been reported in long-distance triathletes [47]. The symptoms of hyponatremia are similar to those associated with dehydration and include mental confusion, weakness and fainting. Such symptoms are usually seen at serum sodium concentrations of 126-130 mmol/L. Below 126 mmol/L, seizures, coma and death may occur [8].

Because the symptoms of hyponatremia are so similar to those of dehydration, that condition may be dangerously misdiagnosed in endurance races athletes. The usual treatment for dehydration is oral and intravenous administration of fluids. If such treatment were to be given to a hyponatremic individual, the consequences could be fatal [8].

Hyponatremia may occur in a state of euhydration or even dehydration, but it is generally associated with fluid overload [47] and the cause is the fluid intake higher than sweat rate, that causes dilutional hyponatraemia [48]. Triathletes may often develop hyponatremia without displaying symptoms [8].

In order to prevent hyponatremia, avoiding overhydration and informing athletes about the potential dangers of drinking too much water are recommended. When compared with water, a sodium-containing drink attenuated the drop in plasma sodium [49]. However, when salt tablets (700 mg/h) were given to ironmen-distance triathletes, there was no evidence that sodium intake significantly influenced changes in plasma sodium concentration or plasma volume more than did fluid replacement alone [50].

## 6. Conclusions

Physiological adaptations to physical exercises lead to blood volume redistribution favoring the working muscle supply with oxygen and energy-yielding substrate as well as the skin for heating dissipation as sweat. Strenuous exercise and/or hot-humid environments precipitate body dehydration, which may induce core hyperthermia, muscle glycogen depletion, gastric emptying delay, gut underperfusion (and ischemia) followed by endotoxemia or anaphylaxis. Rapid fluid delivery from fluids intake is the goal of oral rehydration solutions and sports drinks, that provide the addition of sodium and carbohydrates to assist the intestinal absorption of water and muscle-glycogen replenishment, respectively. However, sometimes, fluid delivery and carbohydrate delivery are difficult to reconcile as carbohydrate-rich beverages decrease fluid delivery to the gut, thus delaying water absorption and accentuating gut underperfusion. It is necessary to inform athletes about potential dangers of drinking too much water, advise them to refrain from using hypertonic fluid replacements.

### Nutritional Recommendations

• During intense exercise, is recommended an intake of 0,5 L/hour of sports beverages.

• A CHO (<10%) and sodium beverage should be encouraged.

• To increase the CHO exogenous oxidation, glucose plus fructose should be consumed.

## Competing interests

The authors declare that they have no competing interests.

## Authors' contributions

EPO wrote the manuscript, revised it and approved the final version of the manuscript. RCB wrote, read and approved the final version of the manuscript.
